# Prevalence of tuberculous lesion in cattle slaughtered in Mubende district, Uganda

**DOI:** 10.1186/s12917-017-0991-x

**Published:** 2017-03-21

**Authors:** Daniel Pakasi Nalapa, Adrian Muwonge, Clovice Kankya, Francisco Olea-Popelka

**Affiliations:** 1 Department of Biosecurity Ecosystem and Veterinary Public Health, College of Veterinary Medicine, Animal Resources and Biosecurity, Makerere University, P.O. Box 7062, Kampala, Uganda; 2 Division of Genetics and Genomics, The Roslin institute, University of Edinburgh, Easter bush Campus, EH259RG Edinburgh, UK; 3 College of Veterinary Medicine and Biomedical Sciences, Department of Clinical Sciences & Mycobacteria Research Laboratories, Colorado State University, Fort Collins, CO USA

**Keywords:** Tuberculous -like lesions, Uganda cattle corridor, Bovine tuberculosis

## Abstract

**Background:**

The aim of this study was to estimate the prevalence of gross pathology suggestive of bovine tuberculosis (TB-like lesions) and evaluate animal’s characteristics associated with the risk of having bovine TB-like lesions among cattle slaughtered in Mubende district in the Uganda cattle corridor.

**Method:**

We conducted a cross sectional study in which 1,576 slaughtered cattle in Mubende district municipal abattoir underwent post-mortem inspection between August 2013 and January 2014. The presence of bovine TB-like lesions in addition to the animal’s sex, age, breed, and sub-county of origin prior to slaughter were recorded. Associations between the presence of bovine TB-like lesions and animal’s age, sex, breed, and sub-county of origin prior to slaughter were initially analysed using a univariable approach with the chi-square test, and subsequently with a multivariable logistic regression model to assess the combined impact of these animal characteristics with the risk of having a bovine TB-like lesion. Additionally, and as a secondary objective, tissue samples were collected from all carcases that had a bovine TB-like lesion and were processed using standard *Mycobacterium* culture and identification methods. The culture and acid fast positive samples were tested using Capilia TB-neo® assay to identify *Mycobacterium tuberculosis* complex (MTC).

**Results:**

Of 1,576 carcasses inspected, 9.7% (153/1,576) had bovine TB-like lesions from which *Mycobacterium* spp and Mycobacterium Tuberculosis Complex (MTC) were isolated in 13 (8.4%) and 12 (7.8%) respectively. Bovine TB-like lesions were more likely to be found in females (OR = 1.49, OR 95% CI: 1.06–2.13) and in older cattle (OR = 2.5, 95% CI: 1.64–3.7). When compared to Ankole cattle, Cross breed (OR = 6.5, OR 95% CI: 3.37–12.7) and Zebu cattle (OR = 2.57, 95% CI: 1.78–3.72) had higher odds of having bovine TB-like lesions. Animals from Kasanda (OR = 2.5, 95% CI: 1.52–4.17) were more likely to have bovine TB-like lesions than cattle from Kasambya.

**Conclusions:**

The findings of study reveals that approximately one in ten slaughtered cattle presents with gross pathology suggestive of bovine TB in Mubende district in the Uganda cattle corridor district, however, we isolated MTC in only 8.4% of these bovine TB-like lesions. Therefore, there is a need to understand the cause of all the other bovine TB-like lesions in order to safe guard diagnostic integrity of meat inspection in Uganda.

## Background

Bovine tuberculosis (TB) is a chronic, infectious disease caused by *Mycobacterium bovis (M. bovis)* [1]. This disease has both public health and socioeconomic implications and in Uganda this disease is of concern particularly in rural communities [2, 3]. Apart from reduced productivity of infected cattle [4] and the possibility of carcass condemnation at slaughter, consumption of infected uncooked meat and/or unpasteurized milk (and milk products) increases the risk of zoonotic transmission of *M. bovis* to humans (zoonotic TB) in these communities [2, 3].

There is no official nationwide bovine TB control policy in Uganda, therefore, routine meat inspection at municipal abattoirs represent the first point of detection of this disease [2, 5]. It is noteworthy that not all animals are slaughtered at municipal abattoirs; therefore there is an unknown proportion of animals slaughtered that do not undergo meat inspection in Uganda. In countries where a nationwide bovine TB control program is in place, detection of infected animals is synergised through annual TB skin testing and routine meat inspection [4, 6]. Slaughter detected TB-like lesions are confirmed at laboratory level through microscopic detection of acid fast bacilli before or after selective growth on media [4]. The cost of definitive diagnosis is reported to increases along the diagnostic cascade with molecular based diagnostic tools being the most expensive [7]. To reduce on this cost, rapid simple and inexpensive new generation assays for detecting members of *Mycobacterium tuberculosis* complex (MTC) including *Mycobacterium bovis, Mycobacterium bovis BCG, Mycobacterium canettii, Mycobacterium africanum, Mycobacterium pinnipedii, Mycobacterium microti, Mycobacterium caprae and Mycobacterium mungi.* have been developed, and one such assays is capilia TB-neo [7, 8]. This assay has been used in countries for this purpose and in Uganda it has been mostly used in the diagnosis of human TB [8, 9].

Under Uganda’s field conditions, where abattoir surveillance is the only point where bovine TB can be identified, it is critical that the quality and integrity of meat inspection at this point is maintained by assuring the process is conducted appropriately and by regularly updating prevalence estimates which ensures optimal workload per *post mortem* inspector [10]. Furthermore, it is essential to understand disease drivers in order to strategically allocate scarce resources. Under such settings this process is usually hindered by the lack of optimal numbers of *M. bovis* caused bovine TB-like lesions in order to make meaningful risk assessment studies. A recent study reported that one in fifty slaughtered pigs with bovine TB-like lesions were due to *M. bovis* in Mubende district. Such a finding is not only considered a spill-over event from cattle to pigs according to the epidemiology of bovine TB but it also inherently suggests a high prevalence of bovine TB in the cattle populations.

This study therefore aimed at estimating the prevalence of bovine TB-like lesions in cattle slaughtered at the Municipal abattoir in Mubende District in the Cattle Corridor in Uganda and to identify factors associated with the presence of bovine TB-like lesions among these cattle. Additionally, we aimed to isolate *Mycobacterium* species from these bovine TB-like lesions.

## Methods

### Study site

**Fig. 1:**
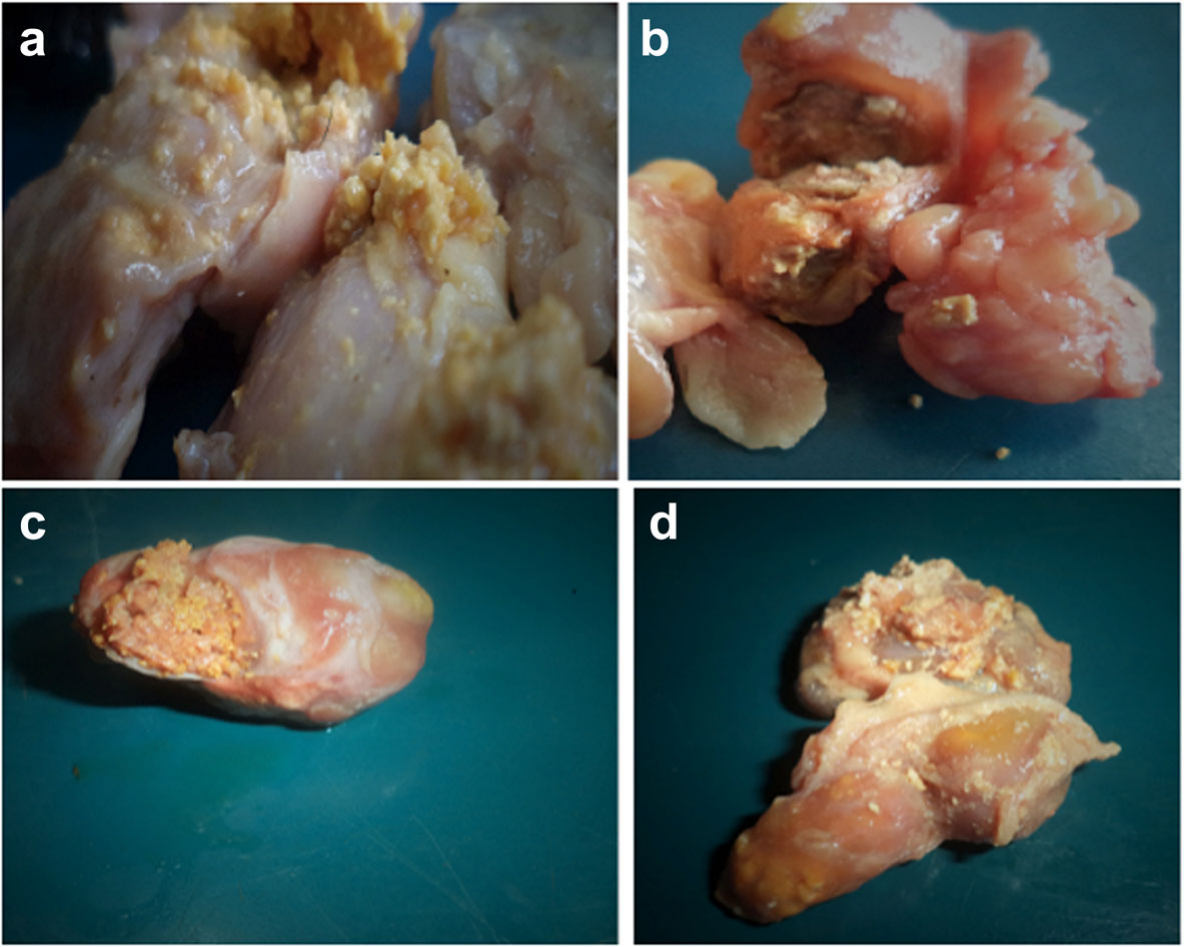
shows a selection of observed BTB-like lesions found in our study. A deep yellow clumped gritty material in mesenteric lymph bodies, **b** **a** tubercle structure from a mediastinal lymph node, **c** & **d** caseous material from part of lymph nodes from the thoracic region

This cross-sectional study was conducted at the municipal abattoir of Mubende district in the Uganda cattle corridor (UCC) between August 2013 and January 2014. The UCC (Fig. 1) is a diagonal expanse of land spanning from the Northeast corner to the south Western corner of Uganda and holds ~ 45% of all the cattle in Uganda [11]. Mubende is located in the centre of the UCC with on average 610,000 and 35,000 human and cattle population respectively [12]. Most of the large herds of cattle are in pastoralist communities that tend to graze cattle communally.

### Sampling and inclusion criteria

A preliminary survey of the study area was carried out in order to secure clearance from the District Veterinary department and to understand the slaughter process in order to develop a sampling strategy that caused the least disturbance to the routine slaughter operations. The sampling sight was the Mubende municipal abattoir located in Mubende town council. All cattle slaughtered at this abattoir between August 2013 and January 2014 were inspected and tissue samples were obtained from all carcasses that presented with bovine TB-like lesions.

### Ante mortem inspection and cattle characteristics

Physical examination of all the animals was conducted to obtain data on age (young and old), sex (male and female), and breed (Ankole-Sanga, East African Zebu, Friesian cross) before slaughter. Age estimation was done using the dental eruption and wear patterns [13]. Animals were classified as young cattle (less than 2.5 years old, and old (greater than 2.5 years old). Additional data on the sub-county of origin prior to slaughter of the animal and total number of animals slaughtered at the abattoir was retrieved from the abattoir records.

### Post mortem inspection

All carcasses of cattle slaughtered during the study period were inspected for the presence of bovine TB-like (tuberculous) lesions. Examination of the carcasses was thoroughly conducted as sanction by the public health Act of Uganda and the World Organisation for Animal Health (OIE) [14]. Briefly, lymph nodes especially parotid, sub maxillary, mandibular, brachial, medial retropharyngeal, mediastinal, hepatic, mesenteric iliac, precrural, prescapular, supramammary, inguinal, and ischiatic lymph nodes, were palpated incised and inspected for the presence of bovine TB-like lesions. Organs including the livers, lungs, kidneys, spleens, intestines, mammary glands were also palpated and incised and inspected for bovine TB-like lesions*.* Lymph nodes that showed the following gross pathological characteristics were collected: increased size, colour changes, and granulation, calcification, and/or caseation texture. Bovine TB-like lesions were collected into a clean, sterile container to avoid contamination with other environmental mycobacteria. The samples were transported at 4 °C to the veterinary public health laboratory where they were stored at −20 °C until they were transferred to Mycobacteriology laboratory in the department of medical microbiology within the school of health sciences at Mulago for mycobacterial culturing.

### Culturing, isolation and identification of *Mycobacterium Tuberculosis Complex* (MTC)

This selective growth was conducted in order to isolate *Mycobacterium spp* from bovine TB-like lesions. Briefly, fat and connective tissue was dissected away from the lymph node; then, 3 g of the tissue was cut into small pieces using a sterile scalpel blade. Tissue samples were crushed in a clean motor using a pestle, the minced tissue was then transferred into a clean, well labelled centrifuge tube. Ten (10) ml of 4% NaOH were added to the minced tissue for decontamination followed by a neutralisation Sterile with PBS (pH 6.8, 0.067 M) added up to the 50 ml mark and the tube was centrifuged at 3200 g for 15 min at 18 °C. All tissue was removed from the centrifuge tube and a phosphate buffered saline was added to 45 ml mark, followed by centrifugation at 3000xg for 15 min at 4° Celsius. The supernatant was carefully poured off leaving only the sediment in the centrifuge tubes. A half (0.5 ml) of the sediment was inoculated into mycobacteria growth indicator tube (MGIT), supplemented with PANTA/OADC and 0.2mls of Lowenstein-Jensen (LJ) supplemented with sodium pyruvate (to promote growth of *M. bovis*). The inoculated MGIT tubes were loaded into the instrument and incubated at 37° Celsius with 5-10% CO2 for up 6 weeks. The media was monitored weekly for growth.

### Acid-fast detection

MGIT tube with growth were examined for acid-fast bacilli by the Ziehl-Neelsen (ZN) by heat fixing the cells on a glass microscope slide and then flooded with carbolfuchsin stain as previously described [15]. The slide was gently and intermitently heated until it steamed for between 2 and 3 min, carbolfuchsin was then poured off and the slide washed thoroughly with water. This was followed by decolourisation with acid-alcohol for five minutes and again washing the slide with water thoroughly. Methylene blue counterstain was then flooded on the slide for one minute and the slide washed with water. Finally excess water was blotted off and the slide dried before viewing under light microscope (Olympus CX31) using a 10^3^ magnification.

### Capilia TB-Neo

The samples that were positive on the acid-fast test were then confirmed to be *Mycobacterium tuberculosis* complex (MTC) using capilia TB-Neo which detects the presence of MPB64 antigens specifically produced by MTC [8]. The kit includes a test plate with a carrier strip composed of a sample placing area, a reagent area including a colloidal gold labelled anti-MPB64 monoclonial antibody and a developing area that fixes the anti-MPB64 monoclonial antibody [7, 8]. Dissolved colony material of a sample (100 μl) was placed on the placing area of the test plate, the colloidal gold labelled anti- MPB64 antibody dissolves and forms an immune complex with MPB64 antigens in the sample. This immune complex migrates through the developing area by capillary action becoming captured by the anti-MPB64 antibody fixed in the developing area, and forms a red purple line of colloidal gold in the reading area. The red purple line visually displays the existence of MPB64 antigens in the sample. Regardless of the existence of the MPB64 antigens in the sample, excess colloidal gold-labelled anti-MPB64 antibody further migrates through the developing area, becoming captured by anti-mouse immunoglobulin antibodies fixed in the developing area, and red purple line in the reading. This means the colloidal gold-labelled anti-MPB64 antibody has migrated normally. Results are interpreted after 15 min.

### Data management and analysis

Individual animal data collected from the abattoir was merged with laboratory results and entered into Microsoft excel 2007. Summary descriptive statistics including proportions and 95% confidence intervals were calculated. The chi-square test (univariable analysis), and multivariable logistic regression analyses were used to evaluate associations between cattle characteristics with the presence of bovine TB-like lesions at slaughter. Variables with a *p* < 0.25 in the univariate analysis were offered manually to the multivariable logistic regression model. The Hosmer-Lemeshow test was used to evaluate the overall model fit. Statistical significance was considered at *p* < 0.05.

## Results

### Animal characteristics

One thousand five hundred and 76 animals were inspected during the 6 months study period at the Mubende district Municipal abattoir. There were more male animals (62%) slaughtered than females, and a greater proportion (86%) of animals were young animals. Ankole-Sanga and East African Zebu accounted for over three quarters of the cattle slaughtered in Mubende district abattoir. Furthermore the highest proportion (35%) of animals slaughtered at this abattoir originated from Madudu sub-county while less than a quarter of the animals came from Mubende town council (Table 1).

**Table 1:** Distribution of bovine TB-like lesions identified in cattle slaughtered at the Mubende Municipal abattoir, Uganda

Animal Characteristics	Inspected	Bovine TB-like Lesions	Risk (95% CI)	*X* ^2^	*P*-value
Total	1576	153	9.7 (8.3,11.3)	-	-
*Sex*					
Male	973	78	8.0 (6.4,9.9)	7.81	0.0052
Female	603	75	12.4 (9.9, 15.3)		
*Breed*					
Cross-breeds	53	18	34.0 (21.5, 48.3)	61.4	<0.0001
Zebu	619	83	13.4 (10.8, 16.3)		
Ankole	904	52	5.8 (4.3, 7.5)		
*Age*					
Young	1356	111	8.2 (6.8, 9.8)	24.5	<0.0001
Old	220	42	19.1 (14.1, 24.9)		
*Origin*					
Madudu	550	52	9.5 (7.1, 12.2)	14.71	0.002
Mubende T/C	210	17	8.1 (4.8, 12.6)		
Kasambya	395	25	6.3 (4.1, 9.2)		
Kasanda	421	59	14.0 (10.8, 17.7)		

* Univariable analysis using the Chi-square test

AUC=0.705, HL(df=8), *p* value= 0.1931

### Prevalence and risk factors associated with the presence of bovine TB-like lesions at slaughter

One hundred and 53 carcasses presented with bovine TB-like lesions. We observed that most of the bovine TB-like lesions were anatomically located in the thoracic region. These were mainly from the bronchial and mediastinal lymph nodes as shown in Fig. 1. Most of these lesions were caseocalcerous and grit-like particles embedded within the deep yellow to brownish coloured lesions. This grit-like texture was particularly felt on slicing through the lesion (Fig. 1).

Overall, 153 (9.7%, 95% CI 8.3–11.3) TB-like lesions were found among the 1,576 carcasses inspected (Table 1). Females had a higher risk (12.4%, 95% CI 9.9–15.3) of presenting with a bovine TB-like lesion compared to males (8.0%, 95% CI 6.4–9.9) (Table 1). Older animals had a higher risk (19.1%, 95% CI: 14.1–24.9) of presenting with bovine TB-like lesions than younger animals (8.2, 95% CI: 6.8–9.8). Cross bred had the highest bovine TB-like lesion (34%, 95% CI: 21.5–48.3), while 13.4% (95% CI: 10.8–16.3) of Zebu cattle and 5.8% (95% CI: 4.3–7.5) of Ankole cattle presented with bovine TB-like lesions. Cattle originating in Kasanda had the highest risk (14%, 95% CI: 10.8–17.7) of presenting with a bovine TB-like lesion, followed by cattle from Madudu (9.5%, 95%CI: 7.5–12.2), Mubende (8.1%, 95% CI: 4.8–12.6), and Kasambya (6.3, 95% CI: 4.1–9.2) (Table 1). When adjusting for the combined effect of all these animal characteristics, results from the multivariable analysis (Table 2) indicate that bovine TB-like lesions were most likely to be observed in females (OR = 1.49, OR 95% CI: 1.06–2.13) than in males, and in older cattle (OR = 2.5, 95% CI: 1.64–3.7) when compared to young animals. When compared to Ankole cattle, Cross bred (OR = 6.5, OR 95% CI: 3.37–12.7) and Zebu cattle (OR = 2.57, 95% CI: 1.78–3.72) had higher odds of presenting with bovine TB-like lesions. Animal from Kasanda (OR = 2.5, 95% CI: 1.52–4.17) were more likely to disclose bovine TB-like lesions than cattle from Kasambya.

**Table 2:** Multivariable logistic regression results and adjusted odd ratios for animal sex, breed, age, and origin for the presence of bovine TB-like lesions among slaughtered cattle in Mubende district

Animal Characteristics	Adjusted Odd Ratio	Odds Ratio 95% CI	*P*-value
*Sex*			
Male	Ref	-	
Female	1.49	1.06–2.13	0.017
*Breed*			
Ankole	Ref	-	
Zebu	2.57	1.78–3.72	<0.001
Cross	6.5	2.7–12.57	<0.001
*Age*			
Young	Ref	-	-
Old	2.5	1.64-3.7	<0.001
*Origin*			
Kasambya	Ref	-	-
Madudu	1.66	1.00–2.76	0.05
Mubende TC	1.23	0.63-2.37	0.54
Kasanda	2.51	1.52-4.17	<0.001

### Presence of *Mycobacterium tuberculosis* complex (MTC) in bovine TB- lesions at slaughter

Bacteria from the genus *Mycobacterium* was isolated from 13 bovine TB-like lesions (Table 3). Given this relatively small sample*.* Laboratory analysis shows that in 13 (8.4%) of 153 bovine TB-like lesions belonged to the genus *Mycobacterium* based on the acid-fast test and MGIT culture. Twelve (7.8%) of the 13 were confirmed to be *Mycobacterium tuberculosis* complex (MTC) using the Capillia TB Neo. Two (1.3%) of the 12 are tentatively identified as *Mycobacterium bovis* based on their growth on MGIT, pyruvated LJ and positive result on Capillia TB Neo. We also recorded an 8.5 and 20% contamination rate on Pyruvated LJ and MGIT liquid culture system respectively (Table 4)

**Table 3:** Distribution of genus *Mycobacterium* among bovine TB-like lesions obtained at slaughter at the Mubende Municipal abattoir

Animal Characteristics	Number of bovine TB-like lesions	+ on ZN &/or Capilia (%)
Total	153	13 (8.5)
Sex		
Female	75	2 (2.66)
Male	78	11 (14.1)
Breed		
Zebu	83	6 (7)
Cross	18	1 (5.6)
Ankole	52	6 (12)
Age		
Old	42	2 (4.8)
Young	111	11 (9.9)
Origin		
Kasanda	59	4 (2.6)
Madudu	52	6 (3.9)
Kasambya	25	2 (1.3)
Mubende TC	17	1 (0.7)

**Table 4:** A comparison of diagnostic methods used to identify the causative agent of bovine TB-like lesions obtained from cattle slaughtered in Mubende Municipal abattoir between

Test outcome	TB-like lesions	Z&N	*Pyruvated L.J.	*MGIT	Capillia
Positive	153	13	2	13	12
Negative	0	140	138	108	1
Contaminated	N/A	N/A	13	32	NA
Total	153	153	153	153	13

## Discussion

The World Organisation for Animal Health (OIE) argues that the control of zoonotic TB in human population must start with controlling bovine TB in cattle populations [14]. In countries like Uganda insight into the magnitude of this health concern can only be achieved through applied utilisation of abattoir inspection data. This study therefore aimed at estimating the prevalence of bovine TB-like lesions at slaughter in Mubende district in the Uganda cattle corridor. Furthermore, we investigated factors associated with the presence of bovine TB-like lesions at slaughter in this area.

Approximately, one in ten slaughtered cattle had a bovine TB-like lesion identified during routine meat inspection. This observed prevalence of gross pathology suggestive of bovine TB is higher than previously reported in other districts like Kampala and Masaka [2, 5, 16]. This difference in prevalence between Kampala the capital of Uganda and this cattle corridor district is most likely a reflection of a “filtration process” where cattle are moved through rural cattle markets into the urban abattoirs (Kampala and Masaka), this process ensures that only cattle with a good body condition are sent to markets where they will fetch a higher price per unit kilogram of beef indirectly selecting for healthier animals [17]. This phenomenon has also been observed in Cameroon and Ethiopia [18, 19]. Although the bovine TB-like lesion prevalence here is comparable to Ethiopia [10, 20, 21], the currently observed prevalence is higher than the 7.3, 6.7 and 6.4% reported in Chad, Burkina Faso and Nigeria, respectively. This scenario in Mubende, could be contributing to the observed scenario with a spill-over into pigs, a phenomenon associated with high transmissions pressure that has been previously reported in Mubende district [15].

Female cattle had a higher risk of having bovine TB-like lesions at slaughter in comparison to males, however, in a higher proportion of males genus *Mycobacterium* was isolated from bovine TB-like lesions. The results regarding MTC isolation should be viewed with caution due to the relatively small number of cultures obtained in this study. However, this finding is likely a reflection of cattle management practices especially with regards to the two sexes in pastoral settings. For example, a typical herd will have fewer bulls but kept them for a long time to maximize their breeding potential for the herd [22, 23], other males are kept as steers which are fattened and sold off for slaughter at an early age [17]. These different management practices are likely to increase the risk of exposure to *Mycobacterium* for males.

Older animals were more likely to have bovine TB-like lesions at slaughter which is in agreement with what has been previously reported in Cameroon and Ethiopia [24, 25]. The odds of cross-bred and Zebu animal having lesions at slaughter were 6.5 and 2.57 times those of an Ankole-Sanga breed, respectively. A similar association with breed has been reported in Cameroon [25]. However, there is a difference in risk status in these two studies, where being a mixed (Cross) breed was a protective factor in Cameroon while in our study in Mubende district cross bred cattle was found to have a higher risk of having bovine TB-like lesions when compared to Ankole cattle. In Cameroon up to 85% of the observed bovine TB-like lesions were due to genus *Mycobacterium* [25] while the opposite was found in this study, with only identifying MTC in 8.4% of bovine TB-like lesions. Animals with different geographical origins prior to slaughter had different risk of having bovine TB-like lesions, and this observation is similar to what had been reported in slaughtered pigs in Mubende [26]. Interestingly, bovine TB-like lesions in the study evaluating pigs in Mubende were mostly caused by non tuberculous mycobacteria [26].

Genus *Mycobacterium* was isolated from only 13 of the 153 lesions most of which belonged to the *Mycobacterium tuberculosis* complex (MTC). This represents 8.4% of MTC detected among all bovine TB-like lesions detected, and although this is lower than what has been reported in Ethiopia and Cameroon [19], these findings are in agreement with previous reports in Uganda [2, 5, 16]. These comparable prevalence estimates in different districts within the Uganda cattle corridor at different time points suggests that indeed the causative agents of bovine TB-like lesions are endemically present in the Uganda cattle corridor. However in-depth microbiological analysis reveals that over 19 per cent of the lesions are due to causes other-than *Mycobacterium*. There is therefore dire need to understand what is causing these bovine TB-like lesions in order to safe guard the integrity of meat inspection as bovine TB detection tool.

From a laboratory diagnostic point of view, these results also reveal a substantial (20%) amount of contamination by other bacteria other than mycobacteria which triggered a much more stringent decontamination protocol on our part. This is reported to have an effect on the survival of mycobacteria in a sample especially those with a low bacillary load [27]. It is therefore possible that the decontamination process in this study reduced our ability to accurately identify/culture *Mycobacterium* spp from the obtained bovine TB-like lesion.

The presence of a large proportion of bovine TB-like lesions caused by other pathogens other than *Mycobacterium* has been reported in Europe [28], but in Africa, in Ghana and Chad 34 and 42% respectively of such lesions were reported to be caused by genus *Mycobacterium* [29, 30]. This has diagnostic implications as such gross pathology in which MTC is not identified, would be bovine TB ‘false positives’ which further compromises the diagnostic utility of the only routinely used public health first line detection of bovine TB. The recovery of members of MTC in isolates is particularly of public health concern as these are known to cause pulmonary and extra-pulmonary TB disease in human population [4]. This is expected to pose a considerable public health risk especially in such pastoral population where there is an overlap with HIV, human-TB, bovine TB and socio-cultural practices that favour *M. bovis* transmission via direct contact with cattle and/or by consumption of animal products (mostly unpasteurized milk products).

Although, the main objective of this study was to investigate prevalence of bovine TB-like lesions (gross pathology) suggestive of bovine TB, we think it is essential to definitively establish if these bovine TB-like lesions are actually caused by *Mycobacterium* spp, and which species (and strains). For this specific study, insufficient funds and resources precluded the option of definitively identifying the *Mycobacterium* species found in these bovine TB-like lesions. Thus, we stored these isolates (alliquotes of DNA), and plan to analyse these samples further to species level. However, the presence of MTC at slaughter highlights the eminent risk of human exposure to bovine TB through foods of cattle origin in communities within the Uganda cattle corridor. This comes at a time when the need to identify zoonotic TB at-risk-communities is now more than ever critical in a bid to pre-empt the impact due to zoonotic TB as countries all over the world lay strategies for a TB free world in 2035 [31].

## Conclusion

The findings in this study reveal that one in ten slaughtered cattle presents with gross pathology suggestive of bovine TB (BTB-like lesions) in Mubende district in the Uganda cattle corridor districts, however we identified MTC in only 8.4% of these lesions. There is need therefore to understand the cause of all the other BTB-like lesions in order to safe guard diagnostic integrity of meat inspection.

## Abbreviations

LJ: Lowenstein-Jensen
MGIT: Mycobacteria Growth Indicator Tube
MTC: Mycobacterium Tuberculosis Complex
OADCOleic: Acid, Albumin, Dextrose, And Catalase
OIE: World Organisation For Animal Health
PANTA: Polymyxin, Amphotericin B, Nalidixic Acid, Trimethoprim And Azlocillin
TB: Tuberculosis
UCC: Uganda Cattle Corridor
UNCST: Uganda National Council For Science And Technology
ZN: Ziehl-Neelsen
